# Distinct patterns of disease activity over time in patients with active SLE revealed using latent class trajectory models

**DOI:** 10.1186/s13075-021-02584-x

**Published:** 2021-07-29

**Authors:** John A. Reynolds, Jennifer Prattley, Nophar Geifman, Mark Lunt, Caroline Gordon, Ian N. Bruce, on behalf of the MASTERPLANS Consortium

**Affiliations:** 1 Rheumatology Research Group, Institute of Inflammation and Ageing, College of Medical and Dental Sciences, University of Birmingham, Birmingham, UK; 2 Rheumatology Department, Sandwell and West Birmingham NHS Trust, Birmingham, UK; 3 Centre for Epidemiology Versus Arthritis, Division of Musculoskeletal and Dermatological Sciences, Faculty of Biology, Medicine and Health, University of Manchester, Manchester Academic Health Science Centre, Stopford Building, Oxford Road, Manchester, M13 9PT UK; 4 Centre for Health Informatics, Division of Informatics, Imaging & Data Sciences, Faculty of Biology, Medicine and Health, University of Manchester, Manchester, UK; 5 Manchester University NHS Foundation Trust, NIHR Manchester Biomedical Research Centre, Manchester Academic Health Science Centre, Manchester, Greater Manchester UK

**Keywords:** Systemic lupus erythematosus, Disease activity, Trajectory modelling clinical trials, Epratuzumab

## Abstract

**Background:**

Systemic lupus erythematosus (SLE) is a heterogeneous systemic autoimmune condition for which there are limited licensed therapies. Clinical trial design is challenging in SLE due at least in part to imperfect outcome measures. Improved understanding of how disease activity changes over time could inform future trial design. The aim of this study was to determine whether distinct trajectories of disease activity over time occur in patients with active SLE within a clinical trial setting and to identify factors associated with these trajectories.

**Methods:**

Latent class trajectory models were fitted to a clinical trial dataset of a monoclonal antibody targeting CD22 (Epratuzumab) in patients with active SLE using the numerical BILAG-2004 score (nBILAG). The baseline characteristics of patients in each class and changes in prednisolone over time were identified. Exploratory PK-PD modelling was used to examine cumulative drug exposure in relation to latent class membership.

**Results:**

Five trajectories of disease activity were identified, with 3 principal classes: non-responders (NR), slow responders (SR) and rapid-responders (RR). In both the SR and RR groups, significant changes in disease activity were evident within the first 90 days of the trial. The SR and RR patients had significantly higher baseline disease activity, exposure to epratuzumab and activity in specific BILAG domains, whilst NR had lower steroid use at baseline and less change in steroid dose early in the trial.

**Conclusions:**

Longitudinal nBILAG scores reveal different trajectories of disease activity and may offer advantages over fixed endpoints. Corticosteroid use however remains an important confounder in lupus trials and can influence early response. Changes in disease activity and steroid dose early in the trial were associated with the overall disease activity trajectory, supporting the feasibility of performing adaptive trial designs in SLE.

**Supplementary Information:**

The online version contains supplementary material available at 10.1186/s13075-021-02584-x.

## Background

Systemic lupus erythematosus (SLE) is a systemic inflammatory autoimmune condition with few licensed treatments. A number of promising agents have failed to show efficacy in phase 3 randomised placebo-controlled trials [1]. These failures are likely multifactorial but may be due, at least in part, to the clinical and serological heterogeneity of SLE and the choice of end-points [2, 3]. Stratified or personalised approaches to treatment is therefore essential for future drug development and for the clinical care of this patient group. Biomarkers that predict response or adverse events are an important unmet need in SLE [1].

In lupus trials, the choice of outcome measure is critical. Measures need to be sensitive to changes in clinical disease activity whilst capturing the breadth of disease manifestations [3]. Composite measures such as the SLE responder index (SRI-4) which comprise objective indices (for example the SLEDAI-2K) along with more subjective measures (e.g. physician global scores) are currently favoured [4]. Whilst the SRI-4 has been used in a number of lupus trials [5, 6], there is evidence that a BILAG-based outcome might be preferable to one based on the SLEDAI score. In the TULIP-1 study of anifrolumab, a greater difference between active drug and placebo was observed using the BILAG-based Combined Lupus Assessment (BICLA) than with the SRI-4 [7].

In other conditions, including rheumatoid arthritis (RA), latent class models have identified trajectories of disease activity. This approach offers the opportunity to identify unobserved groups of patients (latent classes) who display similar changes in disease activity over time. In RA, latent class models have been applied to both clinical trial data [8] and observational cohorts [9–12]. A number of these studies have identified 3 main classes with respect to disease activity; typically, a group that improves rapidly, one that shows little or no improvement and a group that either improves slowly or has an intermediate response [8, 11, 12].

The aim of this study was to identify whether SLE patients follow distinct disease activity trajectories within a clinical trial setting and to identify factors associated with individual trajectories.

## Methods

### Study population

We used data from the 2 multicentre double-blind randomised placebo-controlled trials of epratuzumab (EMBODY™-1 and EMBODY™-2) in patients with active SLE [13]. Our dataset comprised 1202/1584 (75%) of the total cohort and were provided to the MASTERPLANS Consortium by UCB Biopharma SRL, Brussels. Data from the screening visit (up to 14 days prior to randomisation) to the last study visit were included. All patients were required to fulfil the ACR Classification Criteria for SLE and have positive antinuclear antibodies and/or dsDNA antibodies at baseline. Patients with severe active renal or neurological disease were excluded. Patients were randomised to epratuzumab (600 mg weekly or 1200 mg alternate weeks) or placebo in a 1:1:1 ratio in addition to standard of care. Infusions were administered over the first 4 weeks of a 3-month dosing cycle (thus 4 dosing cycles over 48 weeks). Stable doses of immunosuppressant or anti-malarial medication were maintained; dose changes resulted in withdrawal from the study. All patients were required to be taking 5-60mg prednisolone daily (or equivalent) at baseline. Dose increases of up to 25% were permitted from weeks 0–8, with tapering encouraged at a rate of 5mg every 2 weeks aiming for ≤7.5mg day. Follow-up-visits were conducted every 4 weeks until 4 weeks after the end of dosing [13].

### Measurement of disease activity

Disease activity was measured using the British Isles Lupus Assessment Group 2004 (BILAG-2004) [14] and SLE Disease Activity Index 2000 (SLEDAI-2K) [15] indices.

In line with the MASTERPLANS Consortium, which uses BILAG-based analyses across the Consortium datasets, we defined major clinical response (MCR) and improvement at 12 months. MCR was defined as a reduction in all BILAG-2004 index A and B scores to C scores, reduction in steroid dose to ≤ 7.5mg daily and a SLEDAI-2K score of ≤ 4. Improvement was defined as reduction in BILAG-2004 score to no more than one B score with no new domains involved, no increase in steroids or SLEDAI-2K score from baseline.

For trajectory modelling the BILAG-2004 numerical score (nBILAG) was used. The A-E scores of the BILAG-2004 index are converted to numerical values across all 9 domains such that A=12, B=8, C=1, D/E=0 [16].

### Development of latent class mixture models

Latent class mixture models (LCMMs) were used to investigate disease activity trajectories. As it was expected that repeated measures within individuals would be highly correlated, mixed effects models with random intercept were fitted using the “lcmm” function in the *lcmm* package for R with nBILAG as the dependent variable. Models of 1–6 latent classes were constructed using time as linear, quadratic or cubic functions. Models were compared using the Bayesian Information Criteria (BIC) and class size, where >2.5% of the population was required in each class. Model adequacy was measured using the average of posterior probability of assignments [17]. Model development is described in further detail in in the supplementary methods.

The characteristics of patients between latent classes were evaluated using non-parametric tests and multinomial logistic regression models.

### Exploratory PK-PD analysis

Exposure to prednisolone and epratuzumab was estimated using a trapezium model as the sum of the area under the dose-time curve between each time point (see supplementary methods). The absolute number and relative % of CD19^+^ B cells in the PBMC fraction were measured by flow cytometry as previously described [13].

## Results

### Patient characteristics

The dataset comprised 1202 patients with a total of 12,952 study visits. The median (IQR) duration of follow-up within the study was 350 (247,352) days from screening to final visit. Age was provided in 3 bands: <35 years (29.0%), 35–55 years (55.2%) and >55 years (15.8%); ethnicity data was not available. At baseline, 93.9% of patients were receiving oral prednisolone with a median dose of 10 (5, 15) mg/day. The median nBILAG score at baseline was 20 (16, 24) and active disease (defined as a BILAG A or B score) was most frequently present in the mucocutaneous (82.4%) or musculoskeletal (93.7%) domains (Table 1).

**Table 1:** Baseline demographic variables of the cohort analysed

Characteristics	Whole cohort (*n*=1202)	Epratuzumab plus standard of care (*n*=792)	Standard of care only (*n*=410)
Age group (years)			
<35	348 (29.0%)	224 (28.3%)	124 (30.2%)
35–55	664 (55.2%)	436 (55.1%)	228 (55.6%)
>55	190 (15.8%)	132 (16.7%)	58 (14.2%)
Female	1131 (94.1%)	739 (93.3%)	392 (95.6%)
Disease duration (years)	5.58 (1.92, 12.7)	5.75 (1.92, 12.7)	5.46 (1.83, 11.9)
ACR/SLICC-DI	0 (0, 2)	0 (0, 2)	0 (0, 2)
SLEDAI-2K	10 (8, 12)	10 (8, 12)	10 (8, 12)
nBILAG-2004 score median (IQR)	20 (16, 24)	20 (16, 24)	20 (16, 24)
BILAG-2004 A or B system score			
Constitutional	117 (9.73%)	74 (9.34%)	43 (10.5%)
Mucocutaneous	990 (82.4%)	653 (82.5%)	337 (82.2%)
Neuropsychiatric	50 (4.16%)	36 (4.55%)	14 (3.41%)
Musculoskeletal	1126 (93.7%)	741 (93.6%)	385 (93.9%)
Cardiorespiratory	132 (11.0%)	89 (11.2%)	43 (10.5%)
Gastrointestinal	23 (1.91%)	13 (1.64%)	10 (2.44%)
Ophthalmological	15 (1.25%)	10 (1.26%)	5 (1.22%)
Renal	91 (7.57%)	62 (7.83%)	29 (7.07%)
Haematological	12 (1.00%)	9 (1.14%)	3 (0.73%)
Serology			
Anti-Ro	571 (48.2%)	373 (47.8%)	198 (49.0%)
Anti-RNP	353 (29.8%)	235 (30.1%)	118 (29.2%)
Anti-dsDNA	334 (28.2%)	211 (27.1%)	123 (30.4%)
Anti-Smith (Sm)	311 (27.3%)	204 (26.2%)	107 (26.5%)
Low C3 level	395 (33.4%)	257 (33.0%)	138 (34.2%)
Low C4 level	482 (40.8%)	320 (41.1%)	162 (40.1%)
Baseline steroid use	1129 (93.9%)	757 (95.6%)	372 (90.7%)
Baseline steroid dose (mg/day)	10 (5, 15)	10 (5, 15)	10 (5, 15)
Concomitant therapy			
Anti-malarial	884 (73.5%)	595 (75.1%)	289 (70.5%)
Methotrexate	251 (20.9%)	165 (20.8%)	86 (21.0%)
Leflunomide	37 (3.08%)	37 (3.08%)	37 (3.08%)
Azathioprine	321 (26.7%)	321 (26.7%)	321 (26.7%)
Mycophenolate mofetil	143 (11.9%)	88 (11.1%)	55 (13.4%)
Drop out from trial	385 (32.0%)	259 (32.7%)	126 (30.7%)
Withdrawal (lack of efficacy)	174 (14.5%)	115 (14.5%)	59 (14.4%)
Withdrawal (adverse events)	78 (6.49%)	52 (6.57%)	26 (6.34%)

Values are *n* (%) or median (IQR) as appropriate

### Disease activity at baseline and 12 months in the whole cohort

There was no difference in response rates at 12 months using either MCR or improvement definitions between patients receiving epratuzumab or placebo (see supplementary table S1).

Patients with MCR or improvement in the whole cohort (both active treatment and/or standard of care) were older, with lower baseline nBILAG scores, and lower baseline prednisolone doses (*p*<0.001 for each). At baseline, patients with MCR were less likely to have positive anti-dsDNA antibodies (*p*=0.011) or low C3/C4 complement (*p*<0.001, *p*=0.006), and had higher serum creatinine concentrations (<0.001). Patients with improvement had reduced frequency of low C3 levels (*p*<0.001) and were less likely to have anti-RNP antibodies (*p*=0.043). In a sensitivity analysis of patients who received standard of care alone, lower baseline nBILAG, lower prednisolone dose, normal C3 and normal C4 (MCR only) were statistically significant predictors of response (see supplementary table S1).

### Trajectories of numerical BILAG score over time: latent class mixed models

**Fig. 1:**
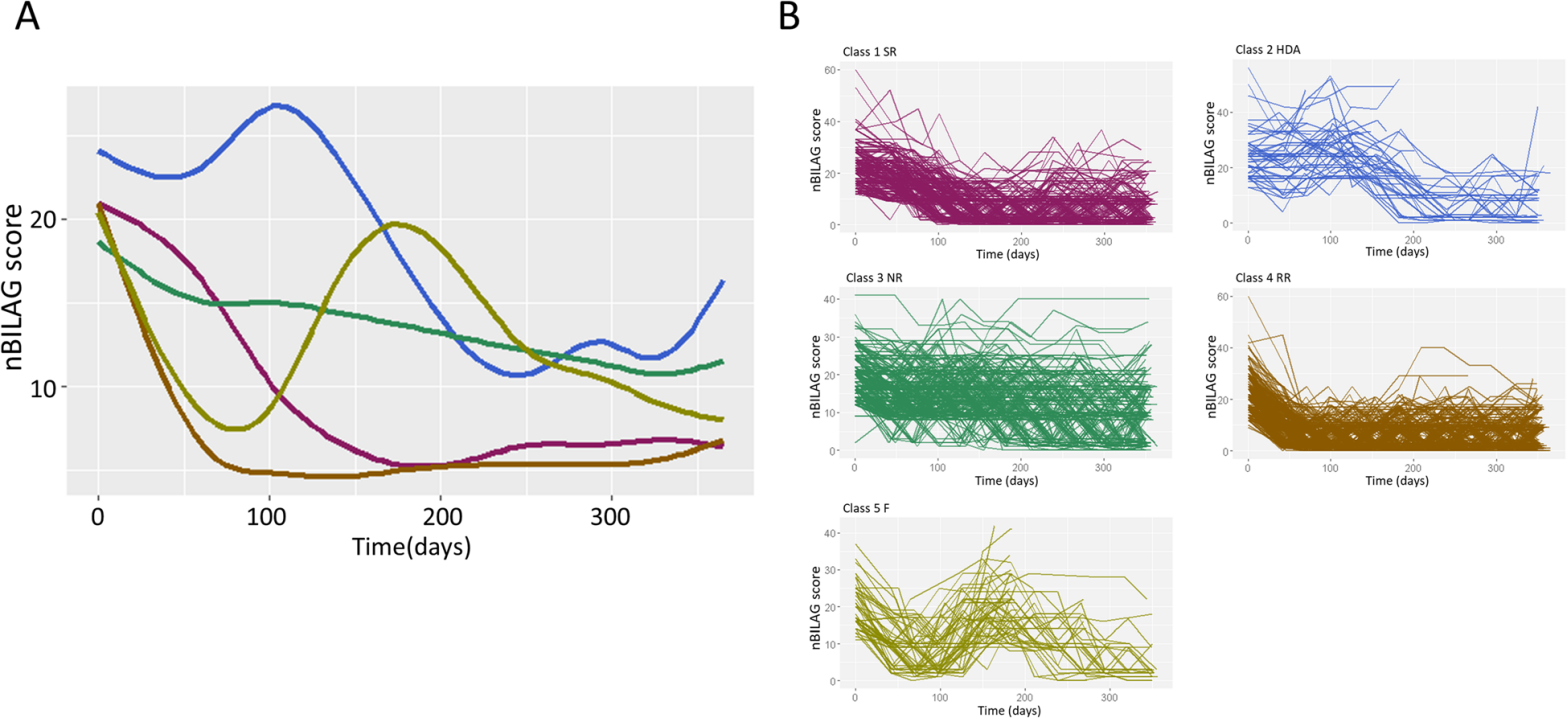
The 5-class cubic LCMM for the whole cohort. **A** The figure shows smoothed curves for each of the 5 classes over time. The trajectories are smoothed using a generalised additive model. **B** The 5 panels show the “spaghetti plots” for each of the 5 classes where each line represents a single patient. SR slow responder, HDA high disease activity, NR non-responder, RR rapid responder, F flare

Latent class mixed models of disease activity over time were constructed for all patients. Numerical BILAG was selected in preference to SLEDAI-2K score as it showed increased sensitivity to change over the first 90 days (see supplementary figure S1). Based on both the BIC and the group sizes, a 5-group cubic spline model was selected, with groups defined by non-responders (NR, 33.9%), slow-responders (SR, 20.7%), rapid responders (RR, 36.7%), high disease activity (HDA, 3.91%) and flare (F, 4.74%) (Fig. 1). The median nBILAG score at baseline and last study visit was 17 (16, 21) and 13 (9, 17) in the NR group, 20 (16, 25) and 3 (1, 10) in the RR group and 20 (16, 25) and 3 (1, 10) in the SR group respectively. The median reduction in nBILAG from baseline to 6 months was 15 (11.5, 19) in the RR group, 15 (12, 19) in the SR group and 5.5 (0, 8) in the NR group (*p*<0.001).

**Fig. 2:**
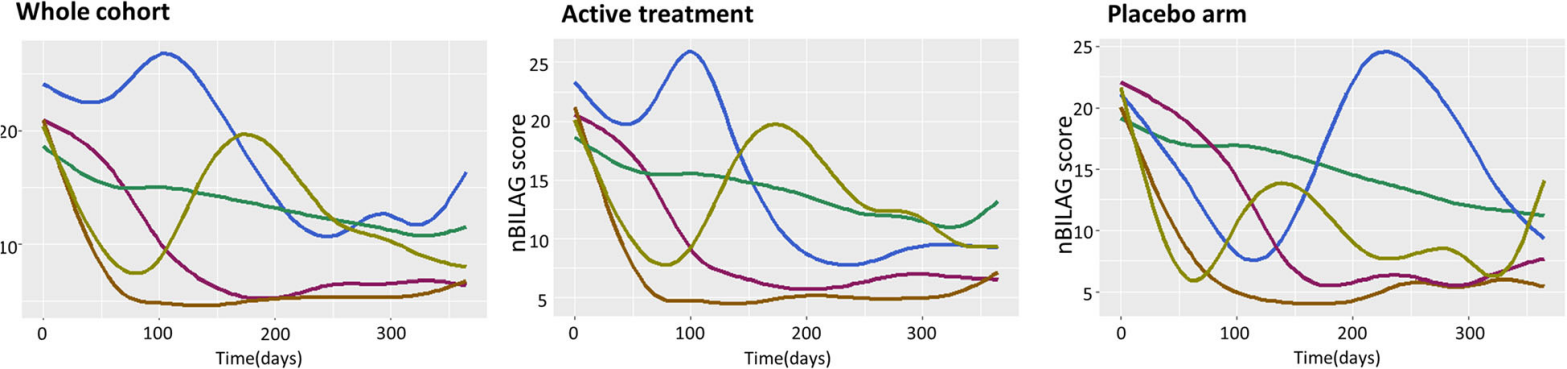
Latent class models according to treatment received. The trajectories in a 5-class model are shown for the whole cohort, those patients receiving epratuzumab (at either dose) or patients receiving placebo respectively. In the epratuzumab group, there are trajectories similar to the whole cohort. In the placebo group, the yellow group flares earlier than in the whole cohort. The HDA group is not observed in the placebo model, but instead a second flare group with a later flare is seen (blue). The 3 trajectories of NR, SR and RR are similar between cohorts, although the RR and SR show greater separation in the placebo group.

**Fig. 3:**
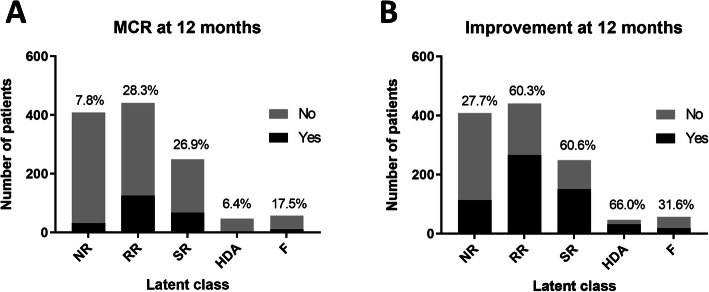
Comparison between latent class membership and response at 12 months. The bars show the number of patients in each of the 5 latent classes and major clinical response (**A**) or improvement (**B**) at 12 months. The number above the bar is the % of patients that meet the MCR or improvement definition in each latent class group. The stricter MCR definition is not met by most of the NR, but also by few of the SR and RR. The definition of improvement would classify more of the patients in the NR latent class as responders

In sensitivity analyses, 5-group cubic spline LCMMs were constructed to include only patients receiving (i) epratuzumab or (ii) placebo. The trajectories of epratuzumab-treated patients were similar to those observed in the whole cohort. The placebo-treated model showed different trajectories with an absent HDA group and two flare groups (less severe, earlier flare and a more severe, later flare) (Fig. 2). A comparison between the latent classes and MCR/improvement at 12 months is shown in Fig. 3 and in the supplementary table S2. The proportion of patients meeting MCR or improvement at 12 months was similar between the RR and SR groups. There was not complete overlap between the trajectories and the MCR/Improvement outcomes in part due a number of patients in each trajectory failing to fulfil the additional SLEDAI and steroid reduction criteria for the landmark end-points (supplementary table S3). The trajectories of SLEDAI score over time, for each of the 5 latent classes, demonstrated similar trajectories (see supplementary figure S2).

As the HDA and F groups each comprise <5% of the population, they were excluded from further analyses.

### Characteristics of responder and non-responder latent classes in the whole cohort

Patients in the NR, SR and RR latent classes were not significantly different in terms of age, gender or disease duration (Table 2). Although the SLEDAI-2K score was similar between groups, NR had a lower baseline nBILAG score compared to the other 2 groups (17 [16, 21] vs. 20 [16, 25] and 20 [16, 25] respectively, *p*<0.001). NRs were less likely to have activity in the constitutional, musculoskeletal or cardiorespiratory systems compared to RR and SR (see Table 3). Whilst there were no differences in the drop-out rate due to adverse events (NR 5.9%, SR 5.6%, RR 6.6%, *p*=0.858), drop-outs due to lack of efficacy were significantly higher in the NR group (NR 20.6%, SR 7.6%, RR 8.4%, *p*<0.001).

**Table 2:** Characteristics of patients in the NR, SR and RR latent classes in the whole cohort

Characteristics	Non-responder/NR (*n*=408) (Class 3)	Slow-responder/SR (*n*=249) (Class 1)	Rapid-responder/RR (*n*=441) (Class 4)	*P*-value
Age group (years)				0.669
<35	119 (29.1%)	72 (28.9%)	117 (26.5%)	
35–55	230 (56.4%)	133 (53.4%)	247 (56.0%)	
>55	59 (14.5%)	44 (17.7%)	77 (17.5%)	
Female	387 (94.9%)	237 (95.2%)	412 (93.4%)	0.542
Disease duration (years)	6.04 (2.17, 13.3)	5.17 (1.92. 12.4)	5.33 (1.75, 12.3)	0.373
ACR/SLICC-DI	0 (0, 2)	0 (0, 2)	0 (0, 2)	0.905
SLEDAI-2K	9.5 (8, 12)	10 (8, 12)	10 (8, 12)	0.394
**nBILAG score**	**17 (16, 21)**	**20 (16, 25)**	**20 (16, 25)**	**<0.001**
BILAG 2004 A or B score				
**Constitutional**	**15 (3.68%)**	**26 (10.4%)**	**59 (13.4%)**	**<0.001**
Mucocutaneous	335 (82.1%)	212 (85.1%)	362 (82.1%)	0.535
**Musculoskeletal**	**365 (89.5%)**	**237 (95.2%)**	**425 (96.4%)**	**<0.001**
**Cardiorespiratory**	**25 (6.13%)**	**25 (10.0%)**	**63 (14.3%)**	**<0.001**
Gastrointestinal	3 (0.74%)	6 (2.41%)	8 (1.81%)	0.203
Ophthalmological	4 (0.98%)	2 (0.8%)	6 (1.36%)	0.766
Haematological	5 (1.23%)	2 (0.80%)	5 (1.13%)	0.875
Serology				
Anti-Ro	198 (48.8%)	108 (44.8%)	207 (47.5%)	0.621
Anti-RNP	109 (26.9%)	61 (25.3%)	142 (32.6%)	0.074
Anti-dsDNA	107 (26.3%)	63 (26.1%)	123 (28.3%)	0.759
Anti-dsDNA level* (IU/ml) (n=314)	291 (181, 456)	261 (175, 491)	291 (185, 544)	0.618
Anti-Smith (Sm)	96 (23.7%)	59 (24.5%)	117 (26.8%)	0.548
Low C3 level	129 (32.3%)	78 (32.0%)	143 (32.9%)	0.966
C3 level (g/l)	1.08 (0.84, 1.30)	1.06 (0.85, 1.27)	1.08 (0.83, 1.28)	0.803
Low C4 level	165 (41.3%)	88 (36.1%)	181 (41.6%)	0.320
C4 level (mg/l)	200 (120, 300)	220 (150, 300)	200 (130, 310)	0.591
Epratuzumab (either dose)	252 (61.8%)	170 (68.3%)	300 (68.0%)	0.100
**Baseline steroid dose (mg/day)**	**9.5 (5, 10)**	**10 (5, 15)**	**10 (5, 15)**	**0.0418**
**Cumulative steroid exposure (mg days)**	**2247.5 (1532.5, 3500)**	**2685 (1750, 4062.5)**	**2360 (1685, 3610)**	**0.0027**
Concomitant therapy				
Anti-malarial	279 (72.8%)	187 (75.1%)	327 (74.2%)	0.795
Methotrexate	84 (20.6%)	55 (22.1%)	94 (21.3%)	0.899
Leflunomide	9 (2.21%)	6 (2.41%)	17 (3.85%)	0.312
Azathioprine	116 (28.4%)	73 (29.3%)	104 (23.8%)	0.158
Mycophenolate mofetil	44 (10.8%)	29 (11.7%)	54 (12.4%)	0.801
**Withdrawal (lack of efficacy)**	**84 (20.6%)**	**19 (7.63%)**	**37 (8.93%)**	**<0.001**
Withdrawal (adverse events)	24 (5.88%)	14 (5.62%)	29 (6.58%)	0.853

Values are *n* (%) or median (IQR) as appropriate

*Only in patients with high-dsDNA

**Table 3:** Multinomial logistic regression models of baseline variables associated with being in either the RR or SR latent class compared to NR using data from the whole cohort

	Slow responder (SR)			Rapid responder (RR)		
	RRR	95% CI	*p*	RRR	95% CI	*p*
Age group						
<35	Ref	Ref	Ref	Ref	Ref	Ref
35–55	0.956	0.665, 1.373	0.806	1.092	0.799, 1.492	0.579
>55	1.232	0.757, 2.001	0.401	0.137	0.868, 2.029	0.191
Female	1.071	0.518, 2.212	0.825	0.771	0.432, 1.137	0.378
Disease duration	0.988	0.969, 1.008	0.255	0.991	0.975, 1.008	0.312
Disease activity						
**Baseline nBILAG-2004 score**	**1.092**	**1.061, 1.124**	**<0.001**	**1.083**	**1.055, 1.112**	**<0.001**
Baseline SLEDAI score	1.035	0.991, 1.080	0.122	1.023	0.985, 1.063	0.233
**BILAG A or B constitutional**	**3.054**	**1.585, 5.889**	**0.001**	**4.047**	**2.257, 7.256**	**<0.001**
**BILAG A or B musculoskeletal**	**2.326**	**1.202, 4.503**	**0.012**	**3.129**	**1.733, 5.649**	**<0.001**
**BILAG A or B cardiorespiratory**	1.710	0.959, 3.049	0.069	**2.552**	**1.572, 4.145**	**<0.001**
Medication						
Study arm						
Placebo	Ref	Ref	Ref	Ref	Ref	Ref
**E-mab 1200mg QoW**	1.300	0.881, 1.920	0.187	**1.394**	**1.003, 1.936**	**0.047**
E-mab 600mg QW	1.362	0.929, 1.997	0.113	1.244	0.895, 1.727	0.194
Epratuzumab (either dose)	1.332	0.954, 1.859	0.092	1.317	0.993, 1.747	0.056
**Baseline steroid dose (mg/day)**	**1.022**	**1.003, 1.041**	**0.022**	**1.028**	**1.011, 1.045**	**0.001**
Serology						
Anti-Ro	0.853	0.619, 1.175	0.330	0.950	0.724. 0.124	0.708
Anti-RNP	0.923	0.641, 1.329	0.668	1.316	0.977, 1.771	0.070
Anti-dsDNA	0.992	0.691, 1.424	0.967	1.105	0.815, 1.497	0.518
Anti-dsDNA titre* IU/ml (*n*=314)	1.000	0.999, 1.002	0.776	1.000	1.000, 1.002	0.246
Anti-Smith (Sm)	1.046	0.721, 1.519	0.810	1.184	0.867. 1.617	0.288
Low C3 level	0.978	0.701, 1.389	0.941	1.029	0.770, 1.375	0.848
C3 level (g/l)	0.806	0.493, 1.317	0.389	0.951	0.628, 1.441	0.814
Low C4 level	0.803	0.578 1.116	0.192	1.015	0.770, 1.337	0.916
C4 level (mg/l)	1.001	0.999, 1.002	0.438	1.001	0.999, 1.001	0.920

*Only in patients with high anti-dsDNA

Multinomial logistic regression models were constructed to identify baseline variables associated with being in the RR or SR class, compared with the NR class (Table 3). Higher baseline nBILAG score and steroid dose was associated with being in both RR and SR latent classes. Similarly, active constitutional and musculoskeletal domains were associated with both classes, whilst an active cardiorespiratory domain was only significantly associated with the RR latent class. Receiving epratuzumab (1200mg QoW) was associated with being in the RR group only (relative risk ratio, RRR 1394 [1.003, 1.936]). There were no baseline serological biomarkers which predicted latent class membership. Similarly there were no differences in the change in anti-dsDNA, C3 or C4 levels from baseline to 3 months, 6 months or study end between latent classes, except in the subgroup of patients with high anti-dsDNA at baseline (see supplementary table S4).

In multivariable models adjusted for age group, gender and disease duration, these associations all remained significant (Table 4). In further exploratory analyses, the RR and SR latent classes were combined into a single responder group (see supplementary table S5 and S6).

**Table 4:** Multinomial logistic regression models of baseline variables associated with being in a SR or RR latent class adjusted for age, gender, disease duration and time in the study

	Slow responder (SR)			Rapid responder (RR)		
	OR	95% CI	*P*	OR	95% CI	*P*
**Baseline nBILAG-2004 score**	**1.100**	**1.067, 1.132**	**<0.001**	**1.089**	**1.060 1.119**	**<0.001**
**BILAG A or B constitutional**	**3.072**	**1.576, 5.990**	**0.001**	**4.196**	**2.323, 7.580**	**<0.001**
**BILAG A or B musculoskeletal**	**2.252**	**1.150, 4,410**	**0.018**	**3.073**	**1.692, 5.580**	**<0.001**
**BILAG A or B cardiorespiratory**	1.607	0.894, 2.887	0.113	**2.461**	**1.510, 4.011**	**<0.001**
Medication						
Study arm						
Placebo	Ref	Ref	Ref	Ref	Ref	Ref
E-mab 1200mg QoW	1.344	0.904, 2.000	0.144	**1.417**	**1.017, 1.975**	**0.039**
E-mab 600mg QW	1.379	0.934, 1.036	0.106	1.237	0.887, 1.726	0.209
**Baseline steroid dose (mg/day)**	**1.028**	**1.008, 1.049**	**0.005**	**1.033**	**1.016, 1.051**	**<0.001**

### Relationship between early changes in disease activity and latent class membership

The changes in nBILAG score between baseline and 1, 2 and 3 month time points, and the final study visit, were compared between the RR, SR and NR latent classes (Table 5 and supplementary figure S3). At 1 month, the median change in nBILAG was similar between NR and SR; at 2 and 3 months, clear differences were observed between the 3 latent classes By the final study visit, there was no difference in nBILAG reduction between RR and SR classes but both of these had around a 2-fold greater reduction in nBILAG compared to the NR class. In the NR trajectory, only 29/408 (7.11%), 32/408 (7.84%) and 26/408 (6.37%) had a reduction in nBILAG scores of <10 at 1, 2 and 3 months respectively. The sensitivity and specificity of a change in nBILAG score of <10 to identify patients in the NR latent class was 93.6% and 65.5% respectively.

**Table 5:** Changes in numerical BILAG-2004 index score between baseline and 1, 2 and 3 months by latent class membership

Latent class	1 month	2 months	3 months	Last visit
Reduction in nBILAG score from baseline (median, IQR)				
NR	0 (0, 7)	2 (0, 7)	3 (0, 7)	7 (1, 11)
SR	0 (0, 5)	7 (1, 10)	11 (7, 14)	15 (11, 19)
RR	11.5 (7, 14)	15 (12, 19)	15 (12, 19)	15 (11, 20)
Number (%) of patients with reduction in daily prednisolone dose				
NR	19 (4.7%)	40 (9.8%)	42 (10.3%)	93 (22.8%)
SR	13 (5.2%)	27 (10.8%)	30 (12.1%)	87 (34.9%)
RR	31 (7.0%)	82 (18.6%)	95 (21.5%)	182 (41.3%)

Values are median (IQR)

### Steroid exposure over time and latent classes

Patients in the NR group had slightly lower baseline prednisolone doses than either SR or RR (see Table 2). Amongst patients who completed the trial, those in the RR group had more rapid tapering although the total reduction was only 1.25 [0, 7.5] mg/day (supplementary figure S4). The median cumulative steroid exposure in the first 90 days was higher in the RR and SR groups (965 [495, 1545] and 980 [500, 1485] mg days) vs. the NR group (761 [500, 1185] mg days) (*p*=0.0391). The NR group did not show any early reduction in their steroid dose whilst the RR patients were more likely to have reduced their steroid dose at 2 and 3 months; *p*<0.001 for both (Table 4 and supplementary figure S4). By the last study visit, steroid reduction was greater in the RR group compared to either SR or NR. In a multivariable logistic regression model (comprising age, gender, disease duration, number of days in the trial, and receiving epratuzumab), steroid exposure in the first 90 days (g days) remained independently associated with being in the RR (RRR 1.46 [1.206, 1.789], *p*<0.001] or SR latent classes (1.34 [1.706, 1.688], *p*=0.009) whilst receiving epratuzumab was not (RRR 1.26 [0.926, 1.703] and 1.27 [0890, 1.011] for RR and SR respectively compared to placebo).

### Exploratory PK-PD analysis

The maximum drug concentration (Cmax) was higher with the 1200mg QoW regimen compared to the 600mg QW regimen (median [IQR] 61.5 [51.6, 71.0] vs. 43.7 [36.3, 52.1] 10^3^ units/ml, *p*<0.0001). Conversely, the exposure (AUC) was lower in the 1200mg QoW regimen compared to 600mg QW (45.0 [29.4, 58.0] vs. 58.3 [39.4, 71.4] 10^6^unit days/ml, *p*<0.0001). As the AUC is dependent on the number of days in the trial, the average exposure/day was determined using AUC/number of days in the trial (see supplementary figure S5).

A higher average exposure (10^3^ unit days/ml) was associated with being in the RR or SR class compared to NR after adjustment for age, gender, disease duration, baseline steroid dose and treatment group: relative risk ratio 1.004 (1.001, 1.007) (*p*<=0.016) for RR and 1.005 (1.002, 1.009) (*p*=0.005) for SR. In a sensitivity analysis using the LCMM of only those patients receiving epratuzumab, this observation remained statistically significant: RRR 1.004 (1.0004, 1.007), *p*=0.025, for RR and 1.005 (1.001, 1.009) for SR compared to NR. There was no significant association between Cmax and responder class (not shown).

There was no significant difference in baseline absolute and % CD19^+^ cells in the RR group compared to the SR or NR groups: 105 (41, 225) vs. 90 (34, 175) and 89 (38, 182), *p*=0.108, for absolute counts and 9 (5, 14) vs. 8 (4, 13) and 8(4, 13), *p*=0.169 for % counts.

**Fig. 4:**
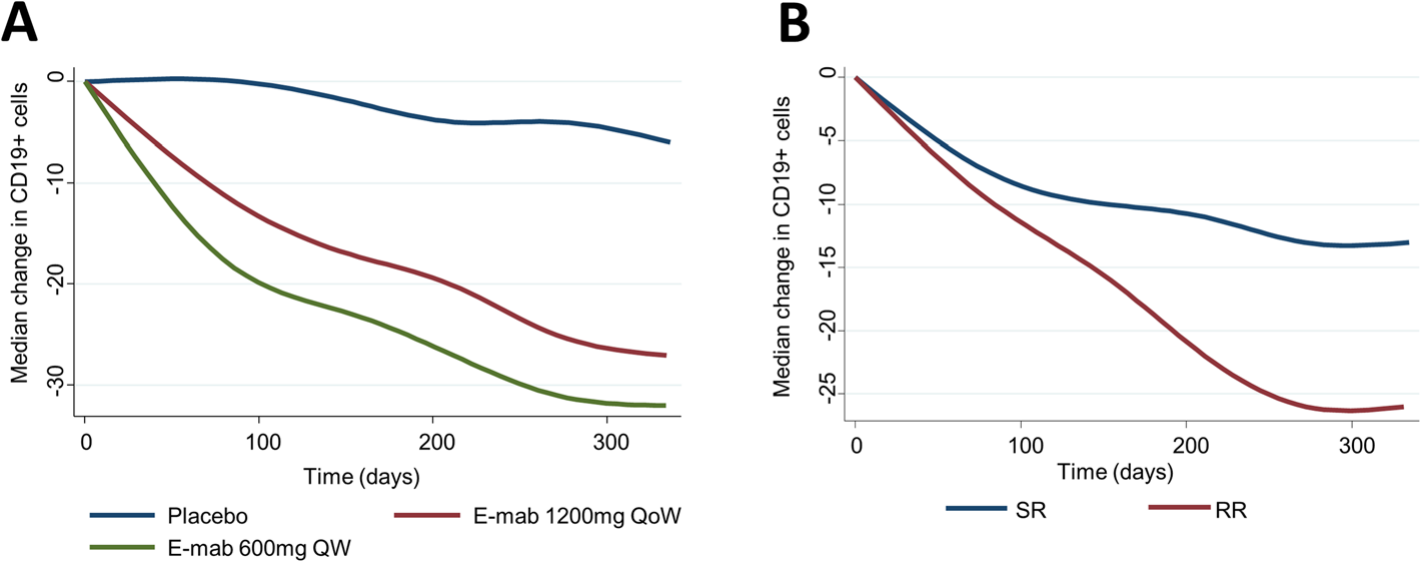
Change in the number of CD19+ cells over the course of the trial. The absolute number of CD19+ cells fell more quickly in the 600mg QW treatment group compared to the 1200mg QoW group (**A**). There was a trend towards a greater reduction in CD19+ cell count in the RR group compared to the SR group (**B**). The graphs show LOWESS smoothed curves

Treatment with epratuzumab reduced CD19^+^ counts (Fig. 4). Using a population-averaged linear model adjusted for age group, gender disease duration and baseline nBILAG score, both epratuzumab doses, were associated with a reduction in CD19^+^ counts (*β* −37.6 [− 53.1, −22.1], *p*<0.0001, for epratuzumab 600mg QW and *β* − 38.1 [− 53.7, − 22.5], *p*<0.001, for epratuzumab 1200mg QoW) compared to placebo. There was no statistically significant reduction in absolute CD19^+^ counts at last study visit in RR (− 19 [− 88, 7.5]) compared to SR (− 16.5 [− 74.5, 8]) or NR (− 11 [− 61, 7]), *p*=0.139.

## Discussion

This study is the first to apply latent trajectory modelling to patients with SLE in a clinical trial setting. Patients with active SLE displayed discrete trajectories of disease activity over time. Although 5 classes were considered to best fit the data, the majority of patients were in 3 classes: non-response (NR), rapid response (RR) and slow response (SR). These separated within the first 90 days of the trial period with several baseline factors associated with response/non-response trajectories.

Disease activity trajectories have been observed in patients with RA in both clinical trials [8] and observational cohorts [8, 11, 12]. The number and shape of latent class trajectories is dependent on the data and outcome measures used [9]. It is essential therefore that the outcome measure is sensitive to change over time. The nBILAG score has a wider range of values than SLEDAI-2K and can detect partial or incomplete responses. Relative to the NR, significant changes in nBILAG occurred as early as 1 month in the RR group and 2 months in the SR group. In the RR group, no further change in nBILAG was observed between 3 months and the last visit. This suggests that changes in disease activity typically occur early in the course of clinical trials. These observations may be specific to epratuzumab, and/or the organ system involved; for example, remission may take >12 months in patients with lupus nephritis [18, 19] resulting in different trajectories in that patient group. These data further support the use of the BILAG-2004 index, and BILAG-based outcome measures, in lupus clinical trials. Furthermore, if our observations are replicated in other cohorts, this would enable the design of adaptive trials in SLE. Our data suggests that a non-response trajectory could be identified as early as 3 months, allowing these patients to be re-randomised to the next mode of action agent, greatly enhancing the efficiency and cost-effectiveness of SLE trials.

The latent class approach categorised patients differently compared to fixed outcome measures at 12 months. Using our Consortium’s response end-points of MCR or improvement, there was no association between receiving active drug and improvement/MCR at 12 months, consistent with the phase 3 epratuzumab (EMBODY) trials [13]. In contrast one of the dosing regimens was associated with being in the RR latent class. This supports the findings of the phase 2 study, and the post hoc analysis of EMBODY (which demonstrated improvement in patients with concomitant Sjogren’s syndrome) [20, 21]. However, our results confirm that baseline anti-Ro antibody status alone cannot identify responders. These data need to be interpreted in the context of both phase 3 RCTs not meeting their primary endpoint (BICLA response). Importantly, there was only a modest association between the latent class groups and the MCR/improvement endpoints. This likely reflects that the latent class models are based on changes in BILAG score alone, whilst the composite endpoints included changes SLEDAI score and in daily steroid dose. Patients in RR or SR classes were equally likely to meet each of the criteria (based on BILAG, SLEDAI and steroid) but not necessarily all three. In addition even the usual BILAG-based and SLEDAI-based composite endpoints do not always show complete agreement, as observed in the phase 3 trials of anifrolumab [7, 22].

Exploratory PK analyses identified that average drug exposure, but not maximum drug level, was associated with response. This supports the observation in the APRIL-SLE study that exposure to atacicept (determined using average trough concentrations) was associated with reduced flare rate [23]. Within our study, there was a marked variation in drug exposure between participants. Optimising drug exposure, where the dose of drug is increased until a target exposure is reached, could be included in future adaptive lupus trials to maximise treatment responses. Whilst our study provides some evidence that epratuzumab may be efficacious in the treatment of SLE, the confounding effects of steroids were significant and further analyses of drug exposure are warranted in clinical trial datasets.

Steroid use remains an important concern in SLE trials. The EMBODY studies allowed generous daily prednisolone doses (up to 60mg a day, with increases of up to 25% during the first 4 weeks). Steroid doses remained relatively static over the trial despite the protocol encouraging steroid reduction [13]. There may therefore be intrinsic differences between centres in how aggressively steroid reduction was undertaken and more recent lupus trials have adopted stricter steroid reduction protocols [22]. Interestingly, both baseline dose and steroid exposure was lower in the NR group and adjusting for this eliminated the association between active drug and response. Accounting for overall steroid exposure, rather than simply, baseline dose or change in dose, is therefore important in lupus trials. Early steroid dose reduction, as observed in the RR group, might also relate to the long-term response trajectory and form part of a future measure to identify those patients following a response trajectory within the first 3 months of a trial.

Identifying predictors of response is an important step towards personalised treatment in SLE. Using fixed outcomes, older age, lower baseline nBILAG scores, normal anti-dsDNA antibodies, serum creatinine and complement levels and lower baseline prednisolone doses were associated with response at 12 months. However, the majority of these predictors applied to both active drug and standard of care groups. The latent class analysis identified that higher disease activity (notably constitutional, musculoskeletal and cardiorespiratory domains), steroid and epratuzumab exposure, but not serological biomarkers were associated with response classes. This discrepancy may arise as both high anti-dsDNA and low C3/4 form part of the SLEDAI-2K score and thus contribute to the definitions of MCR and improvement used in the study. In addition, the number of patients with high anti-dsDNA levels was relatively low as the trial excluded patients with severe active lupus nephritis. Patients with lower baseline disease activity and normocomplementaemia may be more likely to respond to standard of care alone, thus reducing the effect size in a trial setting.

Although latent class trajectory modelling remains an exploratory tool, which can be limited by over-fitting models to the data, all 5 trajectories were deemed clinically meaningful. A principal disadvantage of the approach used is that the nBILAG score removes the ability of the BILAG-2004 index to compare between different organ domains. A further limitation is that the RR and SR latent classes are based solely on changes in disease activity, which may not equate to clinical remission or improvement in patient-reported outcomes. Our dataset comprised patients with moderate-severe disease activity within a clinical trial setting. Notably patients with severe active renal and neurological disease, or significant haematological abnormalities, were excluded. The results therefore may not be generalisable to patients with these manifestations or with a mild flare of disease. Similarly, there were only a small number of disease flares. This is not unexpected in a clinical trial setting and analysis of disease flare in SLE would require a long-term observational cohort study.

The ability to predict which disease activity trajectory a patient is likely to follow would allow more efficient and cost-effective trials. Adaptive trial designs where decisions are based on early changes in disease activity and steroid doses, rather than specified ‘landmark’ endpoints, are an attractive option to explore in SLE. Such studies would ensure that the best use is made of all data collected in trials and that an ‘early fail’ decision could be made with sufficient confidence to allow reassignment of patients to an alternative MOA agent. Our results suggest such an approach is feasible and is worthy of further validation and testing. This approach is likely to be most suited to early phase trials; however, if validated in other studies, it may also have a role in later phase trials, especially those addressing a treatment strategy for SLE.

## Conclusion

Distinct trajectories of disease activity over time in patients with SLE were identified in a clinical trial setting. Changes in disease activity occurred early within the trial which may make adaptive trial designs feasible in SLE.

## Supplementary Information

**Additional file 1:.** Supplementary methods, figures and tables.

## Data Availability

The data that support the findings of this study were proved to the MASTERPLANS Consortium by UCB Biopharma SRL, Brussels, but restrictions apply to the availability of these data, which were used under licence for the current study, and so are not publicly available.
