# Supplementary material for: Distinct patterns of disease activity over time in patients with active SLE revealed using latent class trajectory models
Source: Arthritis Res Ther. 2021 Jul 29;23:203. doi: 10.1186/s13075-021-02584-x (PMC8320218; doi:10.1186/s13075-021-02584-x)
Supplement: Supplementary file 1 — Additional file 1:. Supplementary methods, figures and tables. [file 13075_2021_2584_MOESM1_ESM.docx]

**Supplementary methods: LCMM Development**

**Latent class model development**

We constructed a scoping model using K=3 classes based on data from patients with rheumatoid arthritis {Consortium, 2018 #166; Siemons, 2014 #177;Courvoisier, 2016 #178}. The figure shows standardised residual plots for the 3 classes in a model with no random effects. The curved trajectories of 2/3 of the classes suggests that a non-linear (quadratic or cubic) model is preferable.


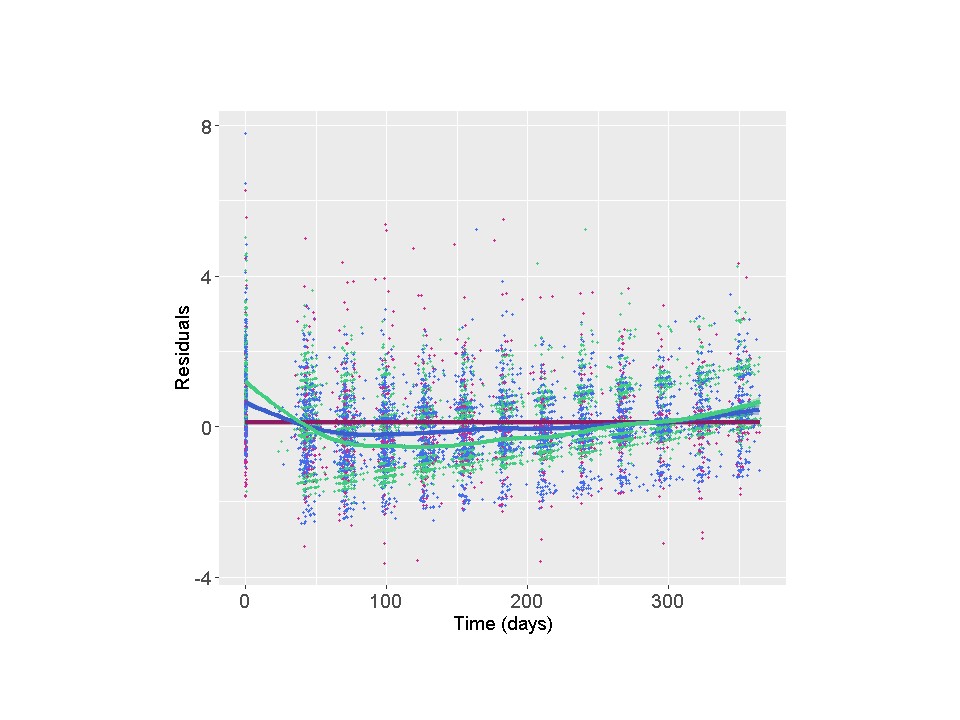


**Refinement of the number of classes:**

We constructed a mixed effects cubic spline model using time^3^ and a linear link function with latent classes K=1-6 using the lcmm function from the *lcmm* package for R. The BIC and sizes of each latent class are presented below. The results from linear and quadratic (time^2^) are shown for comparison.

Although the cubic model with K=6 classes had the lowest BIC, half of the latent classes had a group size of <5%, and so K=5 was selected as the preferred model.

**Table: LCMM Model Characteristics (Whole Cohort Model)**

**Linear**

| Number of Classes | BIC | Number of parameters | Log Likelihood ratio | % of population in each class | | | | | |
| --- | --- | --- | --- | --- | --- | --- | --- | --- | --- |
|  |  |  |  | 1 | 2 | 3 | 4 | 5 | 6 |
| 1 | 81886.2 | 6 | -40921.8 | 100 |  |  |  |  |  |
| 2 | 81907.5 | 9 | -40921.8 | 26.8 | 73.2 |  |  |  |  |
| 3 | 81791.1 | 12 | -40853.0 | 19.5 | 80.5 | 0 |  |  |  |
| 4 | 81756.3 | 15 | -40825.0 | 14.6 | 1.7 | 83.7 | 0 |  |  |
| 5 | 81777.6 | 18 | -40825.0 | 17.1 | 1.8 | 81.1 | 0 | 0 |  |
| 6 | 81798.9 | 21 | -40825.0 | 18.2 | 2.0 | 79.8 | 0 | 0 | 0 |

**Quadratic**

| Number of Classes | BIC | Number of parameters | Likelihood ratio | % of population in each class | | | | | |
| --- | --- | --- | --- | --- | --- | --- | --- | --- | --- |
|  |  |  |  | 1 | 2 | 3 | 4 | 5 | 6 |
| 1 | 79853.1 | 7 | -39901.7 | 100 |  |  |  |  |  |
| 2 | 79233.1 | 11 | -39577.5 | 33.8 | 66.2 |  |  |  |  |
| 3 | 79138.2 | 15 | -39515.9 | 2.1 | 60.1 | 37.8 |  |  |  |
| 4 | 79037.6 | 19 | -39451.4 | 2.2 | 2.2 | 35.9 | 59.7 |  |  |
| 5 | 79012.9 | 23 | -39424.9 | 2.2 | 2.2 | 34.4 | 59.1 | 2.2 |  |
| 6 | 78956.4 | 27 | -39832.5 | 1.8 | 1.7 | 15.7 | 46.7 | 4.0 | 30.0 |

**Cubic Spline**

| Number of Classes | BIC | Number of parameters | Likelihood ratio | % of population in each class | | | | | |
| --- | --- | --- | --- | --- | --- | --- | --- | --- | --- |
|  |  |  |  | 1 | 2 | 3 | 4 | 5 | 6 |
| 1 | 79254.9 | 13 | -39581.4 | 100 |  |  |  |  |  |
| 2 | 78325.9 | 23 | -39581.4 | 41.3 | 58.7 |  |  |  |  |
| 3 | 77946.8 | 33 | -38856.4 | 36.4 | 23.9 | 39.8 |  |  |  |
| 4 | 77716.0 | 43 | -38705.4 | 34.6 | 23.0 | 37.5 | 4.9 |  |  |
| 5 | 77601.3 | 53 | -38612.7 | 20.7 | 3.9 | 33.9 | 36.7 | 4.7 |  |
| 6 | 77525.1 | 63 | -38534.2 | 3.8 | 20.3 | 35.2 | 2.4 | 33.7 | 4.6 |

**Model adequacy assessment**

Model adequacy was determined by assigning each patient to a trajectory class based on the posterior probability. The average of the maximum posterior probability assignments (APPA) was calculated and was >0.7 for all classes in the 5 class model (modes of K=1-6 shown for comparison)

| Number of classes | Average posterior probability assignment (APPA) | | | | | |
| --- | --- | --- | --- | --- | --- | --- |
| 1 | N/A |  |  |  |  |  |
| 2 | 0.887 | 0.921 |  |  |  |  |
| 3 | 0.872 | 0.813 | 0.867 |  |  |  |
| 4 | 0.876 | 0.798 | 0.861 | 0.834 |  |  |
| 5 | 0.819 | 0.877 | 0.832 | 0.856 | 0.817 |  |
| 6 | 0.887 | 0.815 | 0.859 | 0.843 | 0.833 | 0.817 |

**Supplementary methods: Drug exposure and exploratory PK-PD analysis**

Drug exposure was determined for both prednisolone and epratuzumab by calculating the area under the dose-time or concentration-time curves respectively. The trapezium method was used to calculate area (see figure)


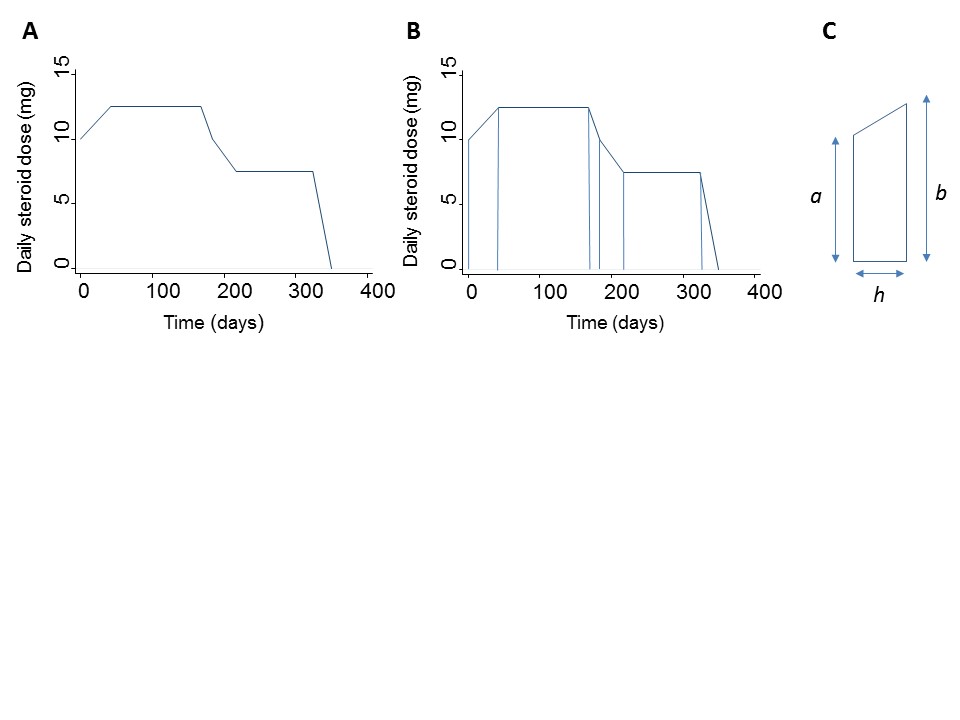


The graphs shows a typical steroid dose-time curve for a single participant (A). This is then converted into trapeziums at each dose change (B). The area of each trapezium is calculated using the formula:

$area= \frac{a+b}{2} \times h$ (C)

The area of the trapezia are then summed to estimate the area under the curve (AUC).

The exploratory PK-PD analysis utilised drug levels which were obtained pre- and post-dose at study visits. The figure below shows examples of the concentration-time plot for patients receiving a 1200mg QoW regimen. To aid understanding solid line has been used although no measurements are taken between data points. Although changes in drug levels over time have been considered to occur in a linear fashion in this model, they are likely exponential. The AUC and Cmax are shown for illustrative purposes.


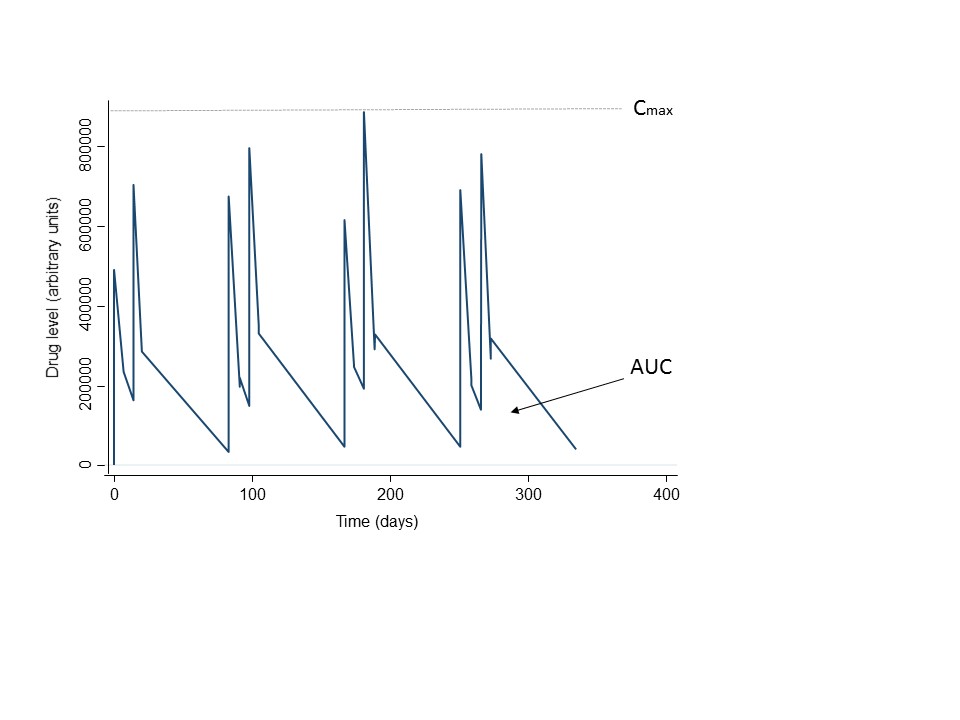


The 1200mg QoW and 600mg QW dosing regimens resulted in differences in the AUC and Cmax. The 1200mg QoW regime (blue) had a lower AUC but greater Cmax than the 600mg QW regime (red). The figures below show k-density plots of AUC and Cmax of patients who received active drug (patients who dropped out of the trial are excluded).


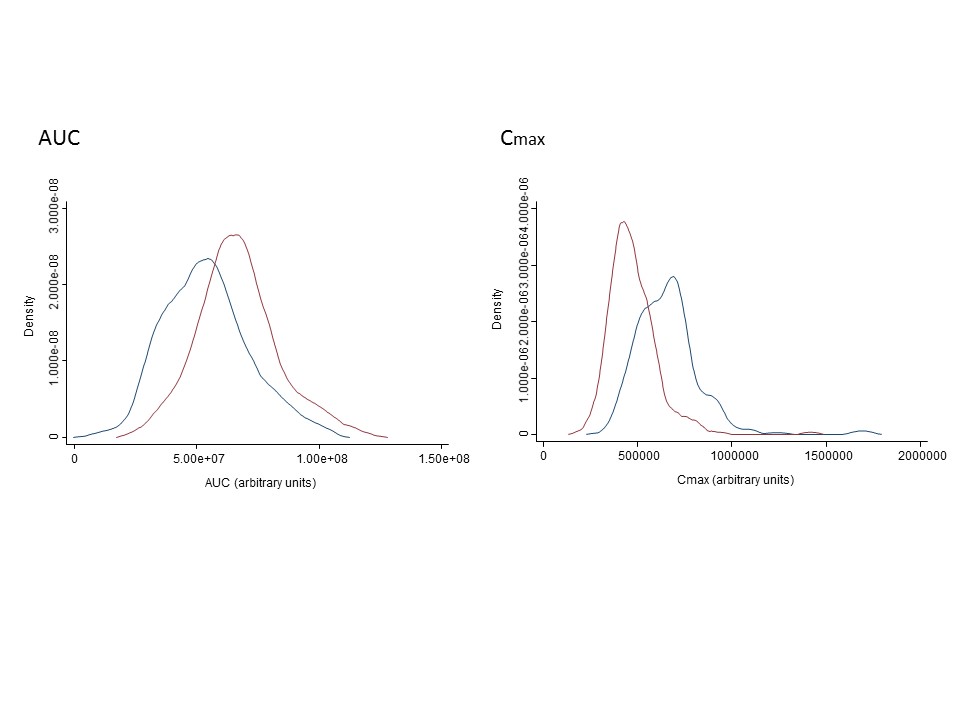


**Supplementary Tables:**

Table S1: Baseline characteristics in patients with Major Clinical Response (MCR) or Improvement at 12 months

Table S2: Comparison of response at 6 and 12 months with 5-group latent class model

Table S3: Univariate logistic regression models of being a “responder” (RR or SR) compared to NR – only patients treated with active drug (i.e no placebo)

**Table S1: Baseline characteristics in patients with Major Clinical Response (MCR) or Improvement at 12 months**

|  | Whole cohort  (n=1202) | Major Clinical Response (12 months) | | | Improvement (12 months) | | |
| --- | --- | --- | --- | --- | --- | --- | --- |
|  |  | Yes (n=237) | No (n=965) | P | Yes (n=564) | No (n=638) | P |
| Gender (male) | 71 | 13 | 58 | 0.759 | 31 | 40 | 0.570 |
| Age band (years)  <35  35-54  **>54** | 348 (29.0)  664 (55.2)  190 (15.8) | 56 (23.6)  122 (51.5)  59 (24.9) | 292 (30.3)  542 (56.2)  131 (13.6) | <0.0001 | 136 (24.1)  314 (55.7)  114 (20.2) | 212 (33.2)  350 (54.9)  76 (11.9) | <0.0001 |
| Drug group  Placebo  1200mgQoW  600mg QW | 410 (34.1)  389 (32.4)  403 (33.5) | 78 (32.9)  84 (35.4)  75 (31.6) | 332 (34.4)  305 (31.6)  328 (34.0) | 0.522 | 182 (32.3)  192 (34.0)  190 (33.7) | 228 (35.7)  197 (30.8)  213 (33.4) | 0.370 |
| Drug group (Emab either dose) | 792 (65.9) | 159 (67.1) | 633 (65.6) | 0.1886 | 382 (67.7) | 410 (64.3) | 0.206 |
| Weight (kg) | 68.4 (58.6, 82.1) | 68.4 (60.0, 82.3) | 68.5 (82.1, 82.3) | 0.6619 | 68.1 (59.0) | 69.0 (58.4, 83.7) | 0.7552 |
| BMI (kg/m2) | 25.7 (21.9, 30.7) | 26.2 (22.0, 30.6) | 25.5 (21.9, 30.7) | 0.5263 | 25.9 (21.9, 30.7) | 25.4 (21.9, 30.6) | 0.662 |
| Disease duration (years) | 5.58 (1.92, 12.4) | 5.00 (1.67, 10.9) | 5.75 (1.92, 12.6) | 0.3024 | 5.58 (1.88, 12.2) | 5.92 (1.92, 12.7) | 0.1893 |
| **Baseline global BILAG score** | **20 (16, 24)** | **17 (16, 21)** | **20 (16, 24)** | **<0.0001** | **17 (16, 22)** | **20 (16, 24)** | **0.0002** |
| Baseline prednisolone dose (mg/day) | 10 (5, 15) | 5 (5, 10) | 10 (5, 15) | <0.0001 | 10 (5, 12.5) | 10 (5, 15) | 0.0001 |
| ACR criteria  Malar rash  Discoid rash  Photosensitivity  Oral ulcers  Arthritis  Serositis  Renal  Neurological  Haematological  Immunological  ANA | 975 (81.1)  388 (32.3)  975 (81.1)  746 (62.1)  1187 (98.8)  393 (32.7)  295 (24.5)  180 (15.0)  731 (60.1)  922 (76.7)  1199 (99.8) | 186 (78.5)  67 (28.3)  181 (76.4)  149 (62.9)  235 (99.2)  80 (33.8)  47 (19.8)  36 (15.2)  146 (61.6)  169 (71.3)  236 (99.6) | 789 (81.8)  321 (33.3)  794 (82.3)  597 (61.9)  952 (98.7)  313 (32.4)  248 (25.7)  144 (14.9)  585 (60.6)  753 (78.0)  959 (99.4) | 0.289  0.089  0.052  0.855  0.532  0.593  0.052  0.940  0.670  0.073  0.792 | 451 (80.0)  171 (34.0)  448 (79.4)  350 (62.1)  559 (99.1)  183 (32.4)  130 (23.0)  75 (13.3)  341 (60.4)  428 (75.9)  561 (99.5) | 524 (82.1)  217 (34.0)  527 (82.6)  396 (62.1)  628 (98.4)  210 (32.9)  165 (25.9)  105 (16.5)  390 (61.1)  494 (77.4)  634 (99.4) | 0.240  0.166  0.237  0.852  0.289  0.978  0.242  0.105  0.538  0.638  0.262 |
| Organ system activity (BILAG 2004 A or B score)  Constitutional  Mucocutaneous  Musculoskeletal  Cardiorespiratory  Gastrointestinal  Ophthalmological  Haematological | 117 (9.73)  990 (82.4)  1126 (93.7)  132 (11.0)  23 (1.91)  15 (1.25)  12 (1.00) | 18 (7.60)  177 (74.7)  226 (95.4)  28 (11.8)  1 (0.42)  2 (0.84)  3 (1.27) | 99 (10.3)  813 (84.2)  900 (93.2)  104 (10.8)  22 (2.28)  13 (1.34)  9 (0.93) | 0.215  0.001  0.235  0.647  0.061  0.532  0.644 | 51 (9.04)  471 (48.8)  534 (55.3)  63 (11,2)  6 (1.07)  4 (0.71)  4 (0.71) | 66 (10.3)  519 (81.3)  592 (92.8)  69 (10.8)  17 (2.67)  11 (1.72)  6 (0.94) | 0.447  0.326  0.179  0.844  0.043  0.114  0.343 |
| **Anti-dsDNA**  Anti-Ro  Anti-RNP  Anti-Smith  **Low C3**  **Low C4**  CRP (continuous) | **334 (27.8)**  571 (47.5)  353 (29.4)  311 (25.9)  **395 (32.9)**  **482 (40.1)**  3 (3, 9) | **50 (21.1)**  100 (42.2)  64 (27.0)  52 (21.9)  **42 (17.8)**  **77 (32.4)**  3 (3, 8.24) | **284 (29.4)**  471 (48.9)  289 (30.0)  259 (26.8)  **353 (36.6)**  **405 (42.0)**  3 (3, 9.11) | **0.011**  0.070  0.382  0.126  **<0.0001**  **0.006**  0.940 | 149 (26.4)  251 (44.5)  **149 (26.4)**  132 (23.4)  **154 (27.3)**  269 (47.7)  3 (3, 7.89) | 185 (29.0)  320 (50.2)  **204 (32.0)**  179 (28.1)  **241 (37.8)**  213 (33.3)  3 (3, 10.9) | 0.346  0.067  **0.043**  0.079  **<0.0001**  0.126  0.122 |

Results are median (IQR) or n (%) as appropriate

Values in red remain statistically significant in a sensitivity analysis of patients who received standard of care only (values not shown).

**Table S2: Comparison of response at 6 and 12 months with 5-group latent class model**

|  | Non-response (NR) | Slow-response (SR) | Rapid-response (RR) | High disease activity (HDA) | Flare (F) |
| --- | --- | --- | --- | --- | --- |
| MCR 6 months (n=171) | 5 (2.92%) | 57 (33.3%) | 109 (63.7%) | 0 | 0 |
| Improvement 6 months (n=495) | 78 (15.8%) | 142 (28.7%) | 264 (53.3%) | 7 (1.41%) | 4 (0.81%) |
| MCR 12 months (n=237) | 32 (13.5%) | 67 (28.3%) | 125 (52.7%) | 3 (1.27%) | 57 (4.74%) |
| Improvement 12 months  (n=564) | 113 (20.4%) | 151 (26.7%) | 266 (47.2%) | 16 (2.84%) | 18 (3.19%) |

**Table S3: Relationship between the 3 principal latent classes and the components of the MCR and Improvement definitions at 12 months**

**Major clinical response (MCR)**

|  | MCR at 12 months | BILAG criterion^1^ | SLEDAI criterion^2^ | Steroid criterion^3^ |
| --- | --- | --- | --- | --- |
| RR | 125 (28.3%) | 246 (55.8%) | 221 (50.1%) | 257 (58.3%) |
| SR | 67 (26.9%) | 133 (53.4%) | 128 (51.4%) | 144 (57.8%) |
| NR | 32 (7.84%) | 76 (18.6%) | 103 (25.3%) | 213 (52.2%) |

1. Reduction in all BILAG-2004 A or B scores to C or D
2. SLEDAI-2K score of ≤4
3. Reduction in daily prednisolone dose to ≤7.5mg

**Improvement**

|  | Improvement at 12 months | BILAG criterion^1^ | SLEDAI criterion^2^ | Steroid criterion^3^ |
| --- | --- | --- | --- | --- |
| RR | 266 (60.3%) | 370 (83.9%) | 357 (80.1%) | 355 (80.5%) |
| SR | 151 (60.6%) | 207 (83.1%) | 204 (81.9%) | 197(79.1%) |
| NR | 113 (27.7%) | 190 (46.6%) | 224 (54.9%) | 288 (70.59%) |

1. Reduction in BILAG-2004 to only 1xB score or better with no new domains involved
2. No increase in SLEDAI-2K score from baseline
3. No increase in daily prednisolone dose

**Table S4: Changes in anti-dsDNA, C3 and C4 complement over time in the NR, SR and RR latent classes**

|  | RR | SR | NR | P value |
| --- | --- | --- | --- | --- |
| Anti-dsDNA* (IU/ml) |  |  |  |  |
| Baseline | 50 (50, 124) | 50 (50, 98) | 50 (50, 103) |  |
| Last visit | 50 (50, 152) | 50 (50, 135) | 50 (50, 122.5) |  |
| Change at 3 months | 0 (0, 0) | 0 (0, 0) | 0 (0, 0) | 0.4732 |
| Change at 6 months | 0 (0, 0) | 0 (0, 4) | 0 (0, 0) | 0.5910 |
| Change at last visit | 0 (0, 0) | 0 (-5, 0) | 0 (0, 2) | 0.5090 |
| Anti-dsDNA (IU/ml) (only dsDNA positive patients at baseline, n=314) | | | | |
| Baseline | 291 (185, 544) | 261 (175, 491) | 291 (181, 456) |  |
| Last visit | 221 (94, 534) | 240 (109, 422) | 238 (102, 478) |  |
| Change at 3 months | -49 (-174, 97) | -35 (-110.5, 111.5) | -2 (-111, 113) | 0.1647 |
| **Change at 6 months** | **-87 (-242, -8)** | **-40 (-107, 133.5)** | **-34 (-143, 58)** | **0.0154** |
| Change at last visit | -61 (-183, 58) | -58 (-158, 100) | -46 (-173, 52) | 0.7698 |
| C3 complement | | | | |
| Baseline | 1.08 (0.83, 1.28) | 1.06 (0.85, 1.27) | 1.08 (0.94, 1.30) |  |
| Last visit | 1.05 (0.83, 1.31) | 1.03 (0.86, 1.27) | 1.07 (0.83, 1.27) |  |
| Change at 3 months | 0 (-0.1, 0.08) | -0.02 (-0.09 0.07) | -0.02 (-0.1, 0.05) | 0.4227 |
| Change at 6 months | -0.02 (-0.11, 0.08) | -0.01 (-0.09, 0.07) | -0.01 (-0.09, 0.07) | 0.8051 |
| Change at last visit | -0.01 (-0.11, 0.09) | -0.02 (-0.11, 0.08) | -0.01 (-0.11, 0.01) | 0.5552 |
| C4 complement | | | | |
| Baseline | 200 (130, 310) | 220 (150, 300) | 200 (120, 300) |  |
| Last visit | 210 (140, 300) | 220 (150, 300) | 210 (130, 300) |  |
| Change at 3 months | 0 (-30, 30) | 0 (-40, 20) | 0 (-30, 20) | 0.1775 |
| Change at 6 months | 0 (-20, 30) | 0 (-30, 20) | 0 (-20, 30) | 0.5509 |
| Change at last visit | 0 (-30, 30) | 0 (-30, 30) | 0 (-20, 30) | 0.1798 |

*lower limit of detection of the assay is 50IU/ml, therefore all values <50 are considered to be 50 for this analysis

**Table S5: Univariate and multivariable logistic regression models of being in the RR/SR latent class compared to NR in the whole cohort**

|  | Unadjusted model | | | Adjusted model* | | |
| --- | --- | --- | --- | --- | --- | --- |
|  | OR | 95% CI | P | OR | 95% CI | P |
| Age group  <35  35-55  >55 | Ref  1.040  1.291 | Ref  0.785, 1.379  0.877, 1.900 | Ref  0.784  0.195 |  |  |  |
| Female | 0.859 | 0.500, 1.475 | 0.582 |  |  |  |
| Disease duration | 0.990 | 0.975, 1.006 | 0.211 |  |  |  |
| Disease activity |  |  |  |  |  |  |
| Baseline nBILAG-2004 score | 1.086 | 1.060, 1.113 | <0.001 | 1.093 | 1.065, 1.121 | <0.001 |
| Baseline SLEDAI score | 1.028 | 0.992, 1.064 | 0.124 | 1.042 | 1.005, 1.081 | 0.028 |
| BILAG A or B constitutional | 3.681 | 2.096, 6.466 | <0.001 | 3.782 | 2.135, 6.700 | <0.001 |
| BILAG A or B musculoskeletal | 2.785 | 1.702, 4.559 | <0.001 | 2.726 | 1.651, 4.502 | <0.001 |
| BILAG A or B cardiorespiratory | 2.239 | 1.410, 3.556 | 0.001 | 2.145 | 1.343, 3.424 | 0.001 |
| Medication |  |  |  |  |  |  |
| Study arm  Placebo  E-mab 1200mg QoW  E-mab 600mg QW | Ref  1.361  1.286 | Ref  1.009, 1.835  0.956, 1.731 | Ref  0.044  0.097 | Ref  1.393  1.286 | Ref  1.027, 1.889  0.951, 1.741 | Ref  0.033  0.102 |
| Epratuzumab (either dose) | 1.323 | 1.024, 1.708 | 0.032 | 1.339 | 1.032, 1.736 | 0.028 |
| Baseline steroid dose (mg/day) | 1.026 | 1.012, 1.042 | 0.001 | 1.031 | 1.015, 1.048 | <0.001 |
| Serology |  |  |  |  |  |  |
| Anti-Ro | 0.914 | 0.715, 1.169 | 0.475 | 0.921 | 0.716, 1.183 | 0.518 |
| Anti-RNP | 1.167 | 0.887, 1.535 | 0.270 | 1.241 | 0.934, 1.650 | 0.136 |
| Anti-dsDNA | 1.064 | 0.806, 1.405 | 0.660 | 1.168 | 0.872, 1.564 | 0.299 |
| Anti-Smith (Sm) | 1.134 | 0.852, 1.510 | 0.388 | 1.201 | 0.889, 1.623 | 0.223 |
| Low C3 level | 1.014 | 0.778, 1.320 | 0.920 | 1.178 | 0.890, 1.560 | 0.252 |
| Low C4 level | 0.934 | 0.727, 1.202 | 0.579 | 1.042 | 0.800, 1.357 | 0.762 |

**Table S6: Univariate logistic regression models of being in the RR/SR latent class compared to NR – only patients treated with active drug (i.e no placebo)**

|  | OR | 95% CI | P |
| --- | --- | --- | --- |
| Age group  <35  35-55  >55 | Ref  1.253  1.336 | Ref  0.873, 1.801  0.825, 2.164 | Ref  0.222  0.238 |
| Female | 1.230 | 0.664, 2.280 | 0.511 |
| Disease duration | 1.002 | 0.983, 1.022 | 0.825 |
| Drop-out from study (all) | 0.491 | 0.352. 0.684 | <0.001 |
| Disease activity |  |  |  |
| Baseline gBILAG score | 1.081 | 1.048, 1.114 | <0.001 |
| Baseline SLEDAI score | 1.021 | 0.976, 1.067 | 0.372 |
| Active constitutional | 4.093 | 1.835, 9.129 | 0.001 |
| Active musculoskeletal | 3.143 | 1.723, 5.735 | <0.001 |
| Active cardiorespiratory | 2.815 | 1.490, 5.321 | 0.001 |
| Medication |  |  |  |
| Baseline steroid dose (mg/day) | 1.010 | 0.992, 1.028 | 0.273 |
| Cumulative steroid exposure (g days) | 1.054 | 0.978, 1.135 | 0.168 |
| Serology |  |  |  |
| Ro | 0.898 | 0.654, 1.232 | 0.505 |
| RNP | 1.252 | 0.878, 1.785 | 0.214 |
| dsDNA | 0.912 | 0.637, 1.306 | 0.616 |
| Smith | 1.141 | 0.786, 1.654 | 0.488 |
| Low C3 | 0.797 | 0.569, 1.115 | 0.186 |
| Low C4 | 0.802 | 0.581, 1.105 | 0.177 |

**Supplementary Figures:**

**Figure S1: Changes in the numerical BILAG score and SLEDAI score over the course of the study**

**Figure S2: Changes in nBILAG score at 1, 2 and 3 months according to latent class membership**

**Figure S3: Median prednisolone dose over time according to latent class membership**

**Figure S4: Relationship between drug exposure (AUC) and time within the trial**

**Figure S5: Relationship between drug exposure (AUC) and time within the trial**

**Figure S1: Changes in the numerical BILAG score and SLEDAI score over the course of the study**


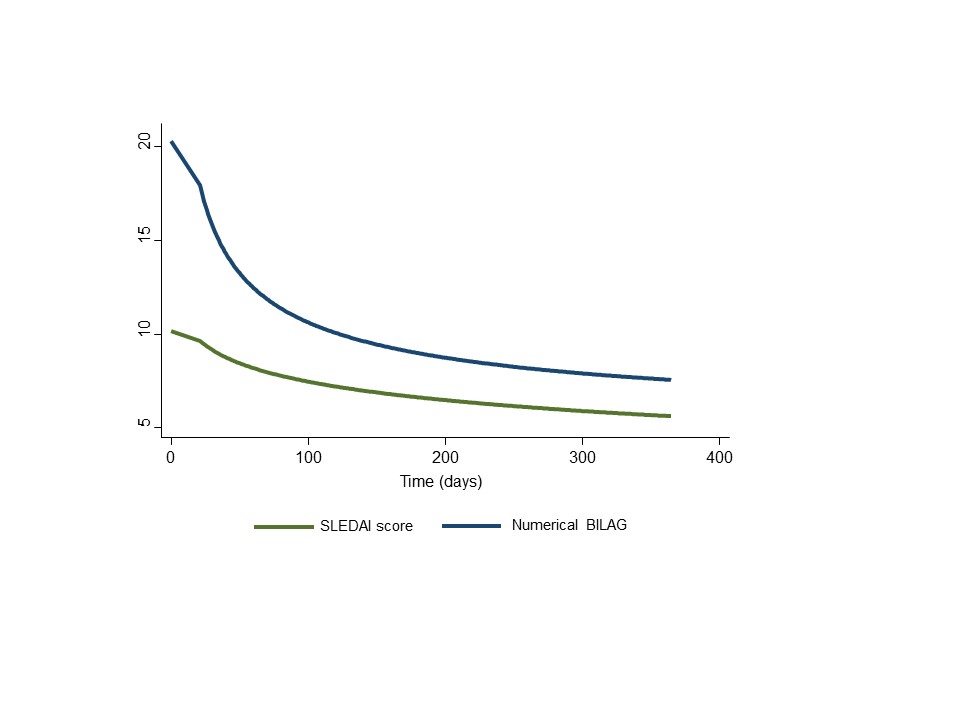


This figure shows the SLEDAI-2K and nBILAG scores over time for the whole cohort (LOWESS smoothed curve). Note that the nBILAG shows the greater sensitivity to change compared to SLEDAI-2K score over the course of the trial.

**Figure S2: Trajectories of SLEDAI score over time for each of the 5 latent classes**


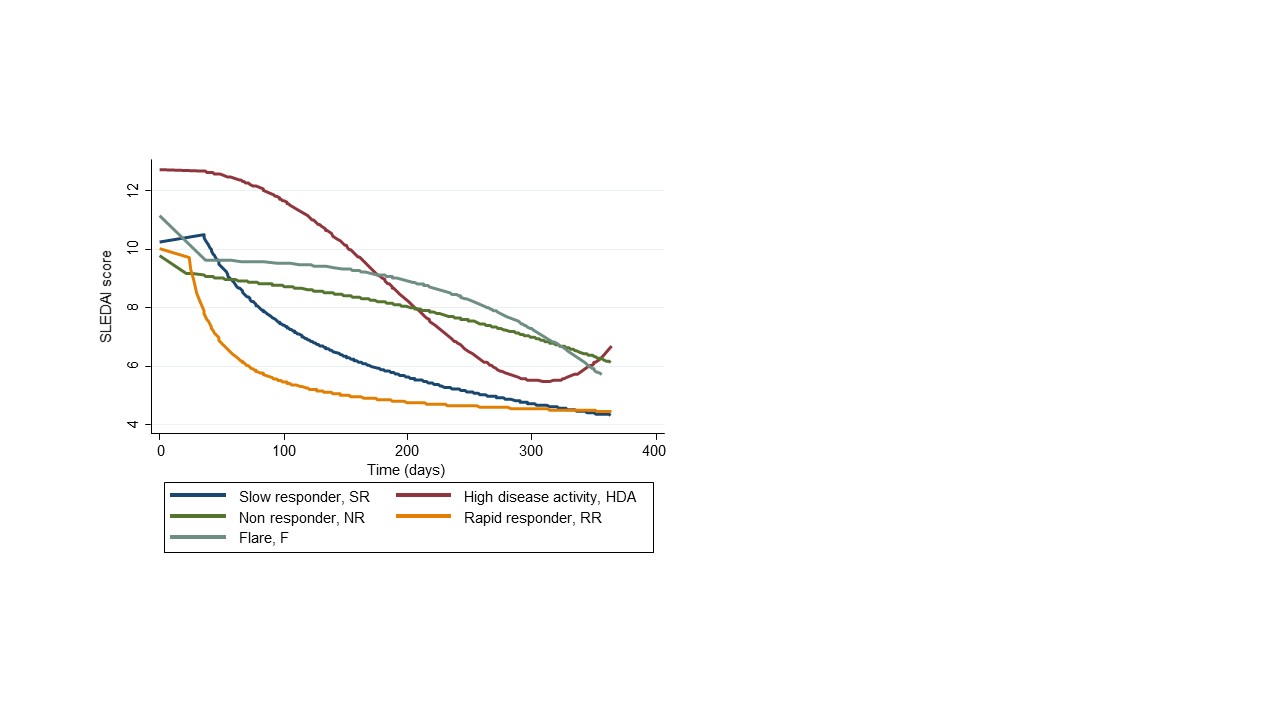


The figure shows the mean SLEDAI score over time for each of the 5 latent classes. The latent class model was developed using the global BILAG score. Fractional polynomial curves were plotted for each of the 5 classes. Overall, the SLEDAI disease activity trajectories of the 3 principal groups (RR, SR and NR) were similar to those in the LCMM. The smaller groups (HAD and F in the LCMM) demonstrated different trajectories.

**Figure S3: Changes in nBILAG score at 1, 2 and 3 months according to latent class membership**

**
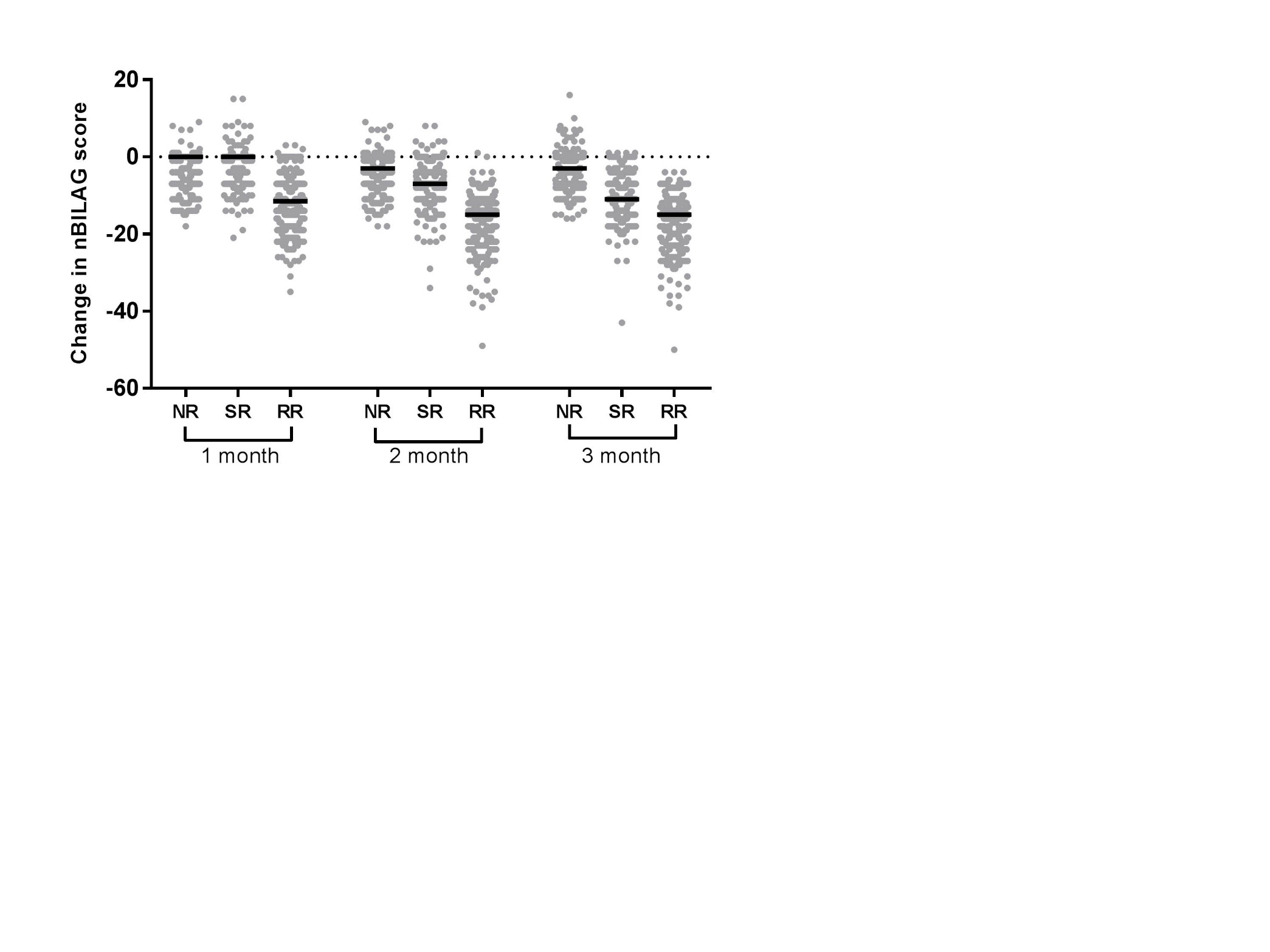
**

Changes in nBILAG score for individual patients at 1, 2 and 3 months compared to baseline, split by latent class membership (see also table in manuscript). At 1 month, there is separation of the RR group compared to NR and SR. By 3 months there is a trend to greater changes in RR > SR > NR.

**Figure S4: Median prednisolone dose over time according to latent class membership**


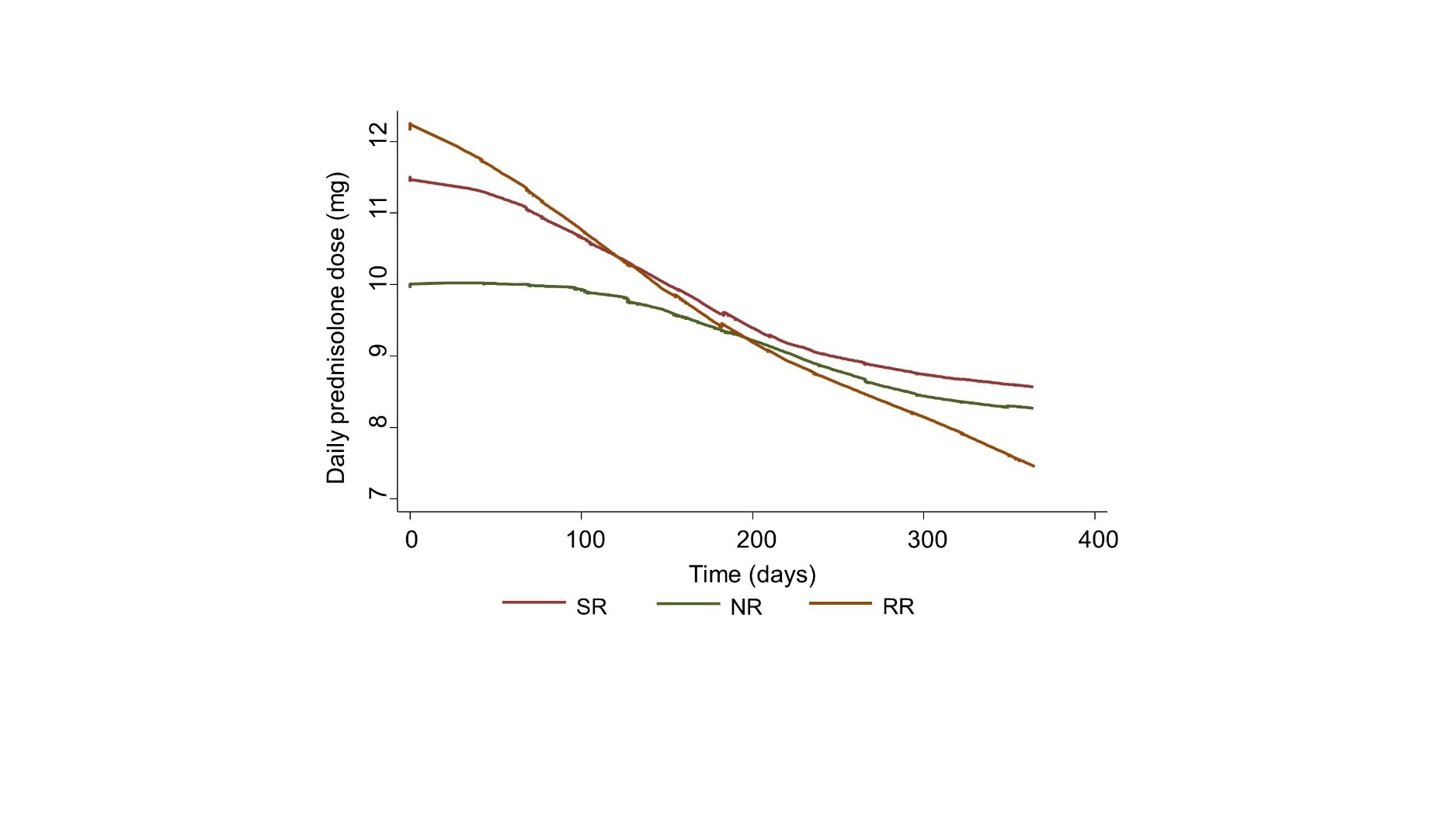


The figure shows LOWESS smoothed curves of the daily prednisolone dose over time for the 3 latent classes. The RR class had higher steroid doses at the beginning of the trial but these were reduced more rapidly than in the SR or RR. The NR had lower steroid doses but little reduction over the course of the study.

**Figure S5: Relationship between drug exposure (AUC) and time within the trial**


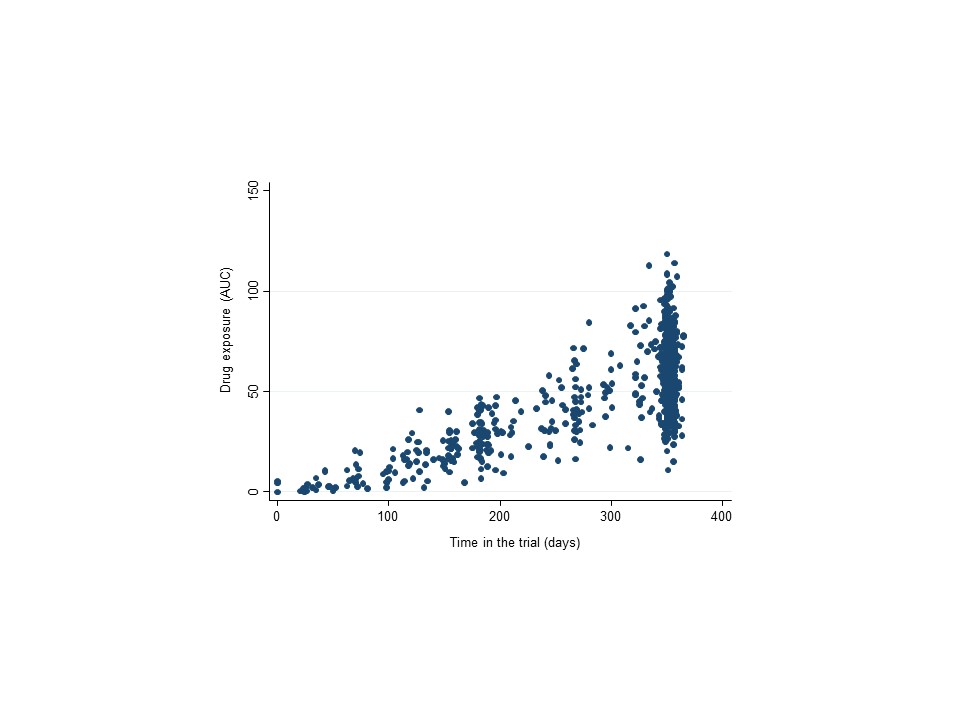


The graph shows the drug exposure as measured by the AUC (in 10^6^ unit days) relative to the number of days the patient remained in the trial (only patients who received Epratuzumab are included). Although a linear relationship is observed, there is significant variability in the amount of drug exposure. For the multivariate analyses, the average exposure was determined by:

$$Average exposue= \frac{AUC}{Time in study (days)}$$
